# Neural sensitivity to the heartbeat is modulated by spontaneous fluctuations in subjective arousal during wakeful rest

**DOI:** 10.1101/2025.03.26.645574

**Published:** 2025-04-01

**Authors:** David Braun, Lotus Shareef-Trudeau, Swetha Rao, Christine Chesebrough, Julia W. Y. Kam, Aaron Kucyi

## Abstract

Spontaneous thoughts, occupying much of one’s awake time in daily time, are often colored by emotional qualities. While spontaneous thoughts have been associated with various neural correlates, the relationship between subjective qualities of ongoing experiences and the brain’s sensitivity to bodily signals (i.e., interoception) remains largely unexplored. Given the well-established role of interoception in emotion, clarifying this relationship may elucidate how processes relevant to mental health, such as arousal and anxiety, are regulated. We used EEG and ECG to measure the heartbeat evoked potential (HEP), an index of interoceptive processing, while 51 adult participants (34 male, 20 female) visually fixated on a cross image and let their minds wander freely. At pseudo-random intervals, participants reported their momentary level of arousal. This measure of subjective arousal was highly variable within and between individuals but was statistically unrelated to several markers of physiological arousal, including heart rate, heart rate variability, time on task, and EEG alpha power at posterior electrodes. A cluster-based permutation analysis revealed that the HEP amplitude was increased during low relative to high subjective arousal in a set of frontal electrodes during the 0.328 s – 0.364 s window after heartbeat onset. This HEP effect was more pronounced in individuals who reported high, relative to low, levels of state anxiety. Together, our results offer novel evidence that at varying levels of state anxiety, the brain differentially modulates sensitivity to bodily signals in coordination with the momentary, spontaneous experience of subjective arousal—a mechanism that may operate independently of physiological arousal.

**Significance Statement:**

Our findings highlight the relationships between spontaneous fluctuations in subjective arousal, brain-body interactions, and anxiety, offering new insights into how interoception fluctuates with changes in internal states. By showing that interoceptive processing is heightened during lower subjective arousal and that this effect is amplified in individuals with higher state anxiety, our study suggests the brain adaptively downregulates interoceptive sensitivity in response to fluctuating internal states. These results have implications for understanding how spontaneous thoughts shape interoception and emotion, particularly in clinical contexts where dysregulated interoception is linked to anxiety and mood disorders. More broadly, our work underscores the need to distinguish between different forms of arousal, advancing understanding of the taxonomy and ways of measuring arousal.

## Introduction

The human brain continually processes signals from the body to guide perception and behavior, a capacity referred to as *interoception* (Engelen et al., 2023). Although early theorists (e.g., James, 1884/1948; Sherrington, 1906) noted its role in cognition and emotion, interoception has recently become a critical focus in cognitive neuroscience (Azzalini et al., 2019; Kluger et al., 2024). A method of measuring interoception—first introduced in the late 20$^{th}$ century (Montoya et al., 1993; Schandry et al., 1986) and now commonly applied with refined approaches (Park & Blanke, 2019)—is the *heartbeat-evoked potential (HEP)*, which involves averaging neural electrophysiological signals time-locked to features of the electrocardiogram (*ECG*) representing the neural processing of heartbeats. Larger HEP amplitude is often interpreted as greater interoception (Judah et al., 2018; Pang et al., 2019; Verdonk et al., 2024), and the HEP has been linked to subjective arousal (Fourcade et al., 2024), a factor central to the present investigation.

Arousal is historically defined as physiological and psychological activation or intensity (Russell, 1980; Russell & Barrett, 1999). Recently, researchers have debated whether arousal is best characterized as unidimensional or multidimensional (Sabat et al., 2025), for example separating subjective and physiological aspects as independent subcomponents (Reid et al., 2024; Satpute et al., 2019), with affective processes often being characterized by subjective arousal. Differences in the HEP have been found in frontal electrodes in the 200–300 ms interval post-heartbeat across various conditions that broadly targeted arousal, including exposure to emotional stimuli (Marshall et al., 2017, 2018, 2020; Sel et al., 2017), food deprivation (Schulz et al., 2015), pain (Shao et al., 2011), and cortisol injections (Schulz et al., 2013). Notably, stimulus- and task-evoked arousal has previously been associated with both increased (Luft & Bhattacharya, 2015) and diminished (Fourcade et al., 2024) interoception.

Another way to target arousal is to measure natural, spontaneous fluctuations during wakeful rest (Chang et al., 2016; Kucyi & Parvizi, 2020), an approach that is also increasingly used to investigate the neural basis of ongoing mental experience (e.g., “spontaneous thoughts” or “mind-wandering”; Christoff et al., 2016; Kam et al., 2021; Shareef-Trudeau et al., 2024; Smallwood et al., 2018). For example, HEP amplitudes vary depending on spontaneous fluctuations in self- or other-referenced thoughts (Babo-Rebelo et al., 2019; Babo-Rebelo, Richter, et al., 2016; Babo-Rebelo, Wolpert, et al., 2016). However, no study to our knowledge has linked the HEP to spontaneous fluctuations in subjective arousal. Spontaneous mental experiences are pervasive in daily life (Killingsworth & Gilbert, 2010), and the arousal component of experience is particularly relevant to mental health conditions involving anxiety (Kucyi et al., 2023).

Anxiety can involve dysregulated interoception, such as heightened or inconsistent sensitivity to bodily signals (Khalsa & Lapidus, 2016). Individuals scoring higher in anxiety tend to exhibit larger HEP amplitudes (Judah et al., 2018; Pang et al., 2019; Verdonk et al., 2024). Given the intimate associations between (i) anxiety and interoception (Janelle, 2002; Noteboom et al., 2001; Roos et al., 2021) and (ii) anxiety and abnormal spontaneous thinking (Fell et al., 2023), spontaneous fluctuations in subjective arousal and their interoceptive underpinnings may prove important for understanding anxiety.

Here we examined the relationship between subjective qualities of ongoing experiences and interoception as well as how this relationship might be influenced by anxiety. Participants completed anxiety surveys and underwent EEG and ECG recordings during a wakeful resting state, intermittently rating subjective arousal via experience sampling. To better understand our “subjective arousal” construct, we assessed whether self-reported arousal converged with physiological measures or was linked to other aspects of ongoing experience. We next leveraged

HEP analysis to examine whether subjective arousal is associated with higher or lower levels of interoceptive processing. Finally, we explored whether anxiety modulated the relationship between HEP amplitude and subjective arousal. Our findings offer important implications for how arousal is conceptualized and how spontaneous internal fluctuations shape the link between interoception and emotion.

## Materials and Methods

### *Participants*

Fifty-four participants (34 female, 20 male) were recruited to participate in this study. Participants were between the ages of 18 and 31, and 50.00% identified as Asian, 35.19% identified as White, 11.11% identified as Black or African American, 1.85% identified as two or more races, and 1.85% identified as other. Participants were recruited both from undergraduate psychology courses for optional course credit and from recruitment posters placed around Drexel and Temple University campuses. Those recruited through the posters received $60 USD of compensation for participation. All participants were fluent in English, had normal or corrected-to-normal vision, were right-handed, and reported no history or current diagnosis of any neurological or psychiatric disorders. All participants provided informed consent for procedures approved by the Drexel University Institutional Review Board. One participant was excluded due to removal of an excessive number of noisy epochs in EEG recordings, one participant was excluded due to EEG recording failure, and one participant was excluded due to behavioral data recording failure, leaving 51 participants for the final analysis.

### *EEG Data Acquisition*

EEG recordings were obtained using a 32-channel Brain Products system, including the BrainAmp MRPlus and the BrainAmp ExG (Brain Products GmbH, Gilching, Germany). We recorded EEG data with the BrainVision Recorder Vers. 1.25.0201 on a Windows operating system using a Dell Latitude 7430 64-bit operating system with a 12$^{th}$ Gen Intel Core i7-1265U and 16GB of RAM. Data were continuously sampled at 5000 Hz. We fitted participants using the BrainCap MR (based on the extended 10/20 system) with embedded carbon wire loops. The system includes

31 scalp electrodes with 10 kOhms of resistance each and an ECG channel with 20 kOhms of resistance. We recorded heartbeat activity via the ECG which was applied to the center of each participant's back as far down as the cord could reach, typically mid-back (Yamashita et al., 2024). Cap setup was concluded when all electrodes' impedance measurements were at levels below 20 kOhms.

### *Mental Health Surveys*

Prior to EEG setup, participants filled out a series of surveys pertaining to their demographic (e.g., age, sex, race) and mental health status. Of these, only the Generalized Anxiety Disorder-7 (GAD-7; Spitzer et al., 2006) and the state scales of the State Trait Anxiety Inventory (STAI-S; Speilberger et al., 1983) were analyzed to capture both trait and state anxiety, respectively, given our focus on the links of anxiety with arousal and interoception (for the complete set of surveys administered in the experiment, see *Table S1*). The state anxiety measure (STAI-S) was a late addition to the experiment after 25 participants' data had been collected. All surveys, except for the STAI-S, were administered online using REDCap electronic data capture tools (Harris et al., 2009, 2019). STAI-S was collected either online or with paper and pencil. Participants were asked to complete all but the state anxiety (i.e., STAI-S) measure prior to arriving for the experiment. Both of the GAD-7 and STAI-S scales have shown high validity and reliability across a wide range of contexts and populations (Gunning et al., 2010; Löwe et al., 2008; Pretorius & Padmanabhanunni, 2023; Vitasari et al., 2011). The GAD-7 and STAI-S are shown to have high internal consistency, both in past research ($\alpha_{GAD} = 0.89$, Löwe et al., 2008; $\alpha_{\mathrm{STAI}} = 0.87$, Spielberger et al., 2017) and in our sample ($\alpha_{GAD} = 0.91$, $\alpha_{STAI} = 0.89$).

### *Experience Sampling*

Throughout the EEG/ECG experiment, participants were intermittently prompted to report on the nature of their ongoing experience using thought probes. Each probe consisted of 13 experience sampling items presented sequentially in a fixed order. For each item, participants provided a rating on a 0–100 slider scale assessing a different dimension of their experience in the moments immediately preceding the probe's onset. The primary experience sampling item relevant to this study measured subjective arousal, asking: *"How activated or energized were you feeling?"* The highest end of the anchor was labeled *"Completely activated"*, while the lowest end was labeled *"Completely deactivated."* Subjective arousal was defined to participants as *"feelings of energy linked to emotion, which may result in heightened bodily energy, tension, or responsiveness",* a definition which is consistent with prior research (Barrett, 1998; Bliss-Moreau et al., 2020; Haj-Ali et al., 2020; Kron et al., 2015; Kuppens et al., 2013; Maher et al., 2019; Nook et al., 2017; Russell, 1980, 2003; Russell & Barrett, 1999). Other relevant experience sampling items for present purposes included probes about future thinking ("Were your thoughts oriented towards the future?" from "Not future oriented" to "Completely future oriented"), deliberate thinking ("How intentional were your thoughts?" from "Completely unintentional" to "Completely intentional"), self-related thinking ("Were your thoughts about yourself?" from "Nothing about you" to "Completely about you "), and difficulty to disengage from thinking ("How difficult was it to disengage from your thoughts?" from "Extremely easy" to "Extremely difficult"). Participants additionally rated their level of confidence in their responses at the end of each thought probe (from "Completely confident" to "Completely unconfident"). For the complete set of experience sampling items used in the experiment, see *Table S2*.

**Procedure**

Upon arrival, participants provided informed consent and were fitted with an EEG cap while receiving task instructions. They were introduced to each experience sampling item and tested for comprehension using a hypothetical scenario in which a student was experiencing stress while preparing for an exam, and participants were asked to respond to experience sampling items as if they were the hypothetical student studying for the exam. The experimenter provided feedback to ensure participant comprehension of instructions before proceeding, and evidence of comprehension was supported by participants giving generally high ratings of confidence in their responses throughout the experiment ($M$ = 17.89, where 0 is most confident and 100 is least; $SD$ = 14.41).

The main task consisted of experience sampling during wakeful rest (see Figure 1A). During wakeful rest, participants sat at a computer and were instructed to “Let your mind wander freely” while focusing on a black screen with a central white fixation cross. At a randomized integer interval every 15–45 s, the fixation cross was replaced by a thought probe with a series of experience sampling items, which participants answered using arrow keys on a laptop computer to adjust a slider scale. After the last item, the fixation cross reappeared, and the cycle repeated.

Prior to the wakeful rest and experience sampling task, participants first alternated between opening and closing their eyes for eight minutes to validate that EEG was recording as expected. Participants next performed the main phase of the experiment, where each cycle of wakeful rest and experience sampling repeated 10 times per run, with 5 total runs, yielding 50 total trials (i.e., thought probe sets). Prior to the start of each task run, the experimenter exited the room. The experimenter reentered the room between each run to give the participants a short break and cue the next task/run. Data collection lasted approximately 60 minutes, with the full session taking about 2 hours.

The first 22 participants were tested in a windowless lab room, which was equipped with a stimulus laptop for task administration and an EEG recording laptop. During the main task, the experimenter visually monitored participants remotely via Zoom (*Zoom Video Communications*, 2024) using a separate monitoring computer. Due to equipment and facility constraints, the remaining 31 participants were tested in a different room that included a glass wall. Participants were seated with their backs to the glass wall and, to minimize external distractions during the task, participants wore earplugs.

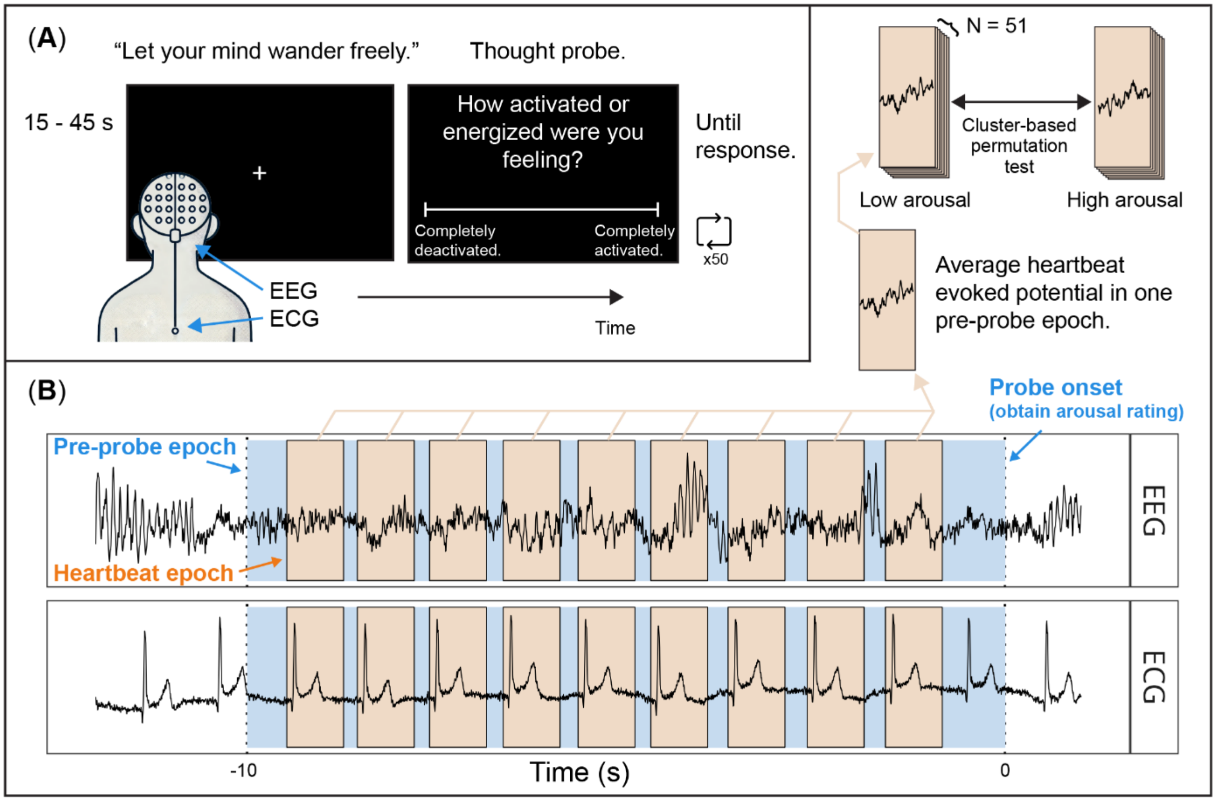

*Figure 1.* Trial sequence and EEG/ECG analysis approach. (A) Participants were instructed to "Let your mind wander freely" while focusing on a black screen with a central, white fixation cross. EEG and ECG were simultaneously recorded throughout. After 15 – 45 s, a series of thought probes appeared, prompting participants for ratings about their experience. This trial sequence was repeated 50 times throughout the experiment. (B) Pre-probe epochs were first defined in the window 10 s prior to thought probe onset. Within pre-probe epochs, heartbeat epochs were defined in the EEG data from -0.1 – 0.65 s around each R-peak in the ECG data. EEG data were averaged over heartbeat epochs across a within-participants median split in subjective arousal ratings separately for each participant. The participant-level averaged data were submitted to a cluster-based permutation analysis to test for significant differences in HEP amplitude depending on subjective arousal level across timepoints within the heartbeat epoch and EEG channels. Note that the figure portrays one example EEG channel, but this procedure was replicated across all 31 EEG channels.

## EEG Preprocessing

Electrophysiological data preprocessing was performed using custom Python scripts based on the MNE library (Gramfort et al., 2013; Larson et al., 2023). Data were downsampled to 250 Hz, bandpass filtered between 1 and 55 Hz, and referenced to the common average. Initial artifact removal was conducted using Extended Infomax independent components analysis (ICA; Lee et al., 1999), with automatic classification and rejection of non-brain artifacts via ICLabel (Pion-Tonachini et al., 2019). Noisy channels were identified via manual inspection and interpolated on the pre-ICA data. Five participants had one channel interpolated, two participants had two channels interpolated, and two participants had three or four channels interpolated. A second ICA decomposition was then performed, and ICLabel results were manually reviewed for further artifact rejection. We chose not to perform baseline correction for HEP analyses because, unlike in stimulus-evoked designs, heartbeat-evoked designs lack a true baseline. Cardiac activity is cyclical, and electrical activity before a heartbeat can reflect both the preceding heartbeat and preparatory processes for the upcoming one (Azzalini et al., 2019).

For HEP analyses, R-peaks in the ECG signal were detected using MNE's preprocessing library. We defined the 10 s period prior to the onset of the thought probe as the time period of interest, as brain states linked to self-report probes can emerge up to 10 s before probe onset (Christoff et al., 2009; Kucyi et al., 2024). Heartbeats occurring within 10 s prior to each thought probe onset were retained for analysis. Following prior work (Petzschner et al., 2019), heartbeats within 700 ms of each other were excluded to prevent overlapping epochs. Within this 10 s pre-probe period, epochs were defined on the EEG channels from -100 ms to +650 ms relative to each

R-peak. Noisy epochs were automatically identified with the autoreject library (Jas et al., 2017), and all epochs were manually inspected before removal.

The cardiac field artifact (CFA) is noise introduced into the EEG signal due to electrical activity from the heart. While ICA is a powerful method for removing many types of artifacts, including the CFA, from EEG (Debener et al., 2010; Devuyst et al., 2008; Hoffmann & Falkenstein, 2008), no known method can entirely remove the CFA from EEG data. To ensure the CFA was not impacting the EEG signal differently across experimental conditions, we contrasted ECG metrics—including heartrate and peak ECG amplitude—across subjective arousal conditions that were of interest in our main analysis (Petzschner et al., 2019).

For spectral analyses involving parieto-occipital alpha power, epochs were defined over the full 10 s period prior to thought probe onset. Noisy epochs were again removed by manually inspecting results from the autoreject library. We examined parieto-occipital alpha power, as past studies have shown a connection between alpha power in this region and various types of arousal (Hofmann et al., 2021; Lozano-Soldevilla, 2018; Schubring & Schupp, 2021; Tuladhar et al., 2007). To examine parieto-occipital power, the EEG signal was decomposed into time-frequency dimensions via MNE's Morlet wavelet transform algorithm with 50 logarithmically spaced frequencies from 2 to 30 Hz and a variable number of cycles per wavelet ($n_{\text{cycles}} = \text{freq}/2$) for occipital and parietal channels only, namely O1, O2, Oz, P3, P4, P7, P8, and Pz (Fourcade et al., 2024). We decomposed the signal from 2-30 Hz to allow for exploratory flexibility in examining power across multiple frequency bands, though our primary focus in this study is on parieto-occipital alpha power.

## Experimental Design and Statistical Analysis

### *Subjective Arousal, Physiological Arousal, and Other Experience Sampling Measures*

To assess the relationship between physiological and subjective arousal, measures of the two constructs were compared. Measures of physiological arousal included heart rate, heart rate variability (HRV), time-on-task, and parieto-occipital alpha power amplitude (Hofmann et al., 2021; Lozano-Soldevilla, 2018; Schubring & Schupp, 2021; Tuladhar et al., 2007), each of which except for time-on-task were summarized over the 10 s period prior to probe onset. Time-on-task, calculated as the probe (i.e., trial) order number, was included because of its well established links to self-reported fatigue, impaired task performance, and negative association with physiological arousal measures (Gergelyfi et al., 2015; Herlambang et al., 2019; Karthikeyan et al., 2021, 2022; Matuz et al., 2019; Melo et al., 2017; Qin et al., 2021; Shi et al., 2022; Zhao et al., 2012). HRV was calculated using the root mean square of differences between successive heartbeats (Shaffer & Ginsberg, 2017). Subjective arousal was measured by self-reported levels of arousal obtained at each thought probe. Within participants, heart rate, HRV, and time on task were each correlated with subjective arousal across trials. The resulting distributions of correlations were Fisher transformed to unbounded space and evaluated with one-sample $t$ tests ($\mu = 0$). Parieto-occipital alpha power (between 8 – 13 Hz) amplitude was averaged across data points with which subjective arousal fell above and below a median split for each participant and compared using a paired-samples $t$ test. We also examined the degree to which ratings of subjective arousal (0-100) were related to other self-report ratings by computing within-participant correlations between subjective arousal and other self-reported experience sampling measures as specified above. These distributions were also Fisher transformed to unbounded space and evaluated with one-sample $t$ tests ($\mu = 0$).

### *Cardiac Interoceptive Sensitivity and Subjective Arousal*

Cardiac interoceptive sensitivity was analyzed in relation to subjective arousal through multiple stages of averaging. Each probe (i.e., trial) was first classified as high or low arousal based on a within-participant median split on arousal ratings. A median split was used to generate dichotomized data for the cluster-based permutation test (see below; for participant-level distributions of subjective arousal responses, see *Figure S1*). For each participant, heartbeat epochs were averaged across each pre-probe period, followed by averaging across the high and low arousal conditions. Consistent with previous work (Al et al., 2021; Fourcade et al., 2024; Tanaka et al., 2023), only time points from 250 ms post-R peak were analyzed to avoid overlap with the CFA. This produced one time (+250 ms to +650 ms) by channel (31 EEG electrodes) array of average voltage measurements for each arousal condition and participant (see *Figure 1B*). Differences between the arousal conditions were evaluated by submitting these arrays to a non-parametric cluster-based permutation analysis (Maris & Oostenveld, 2007), with a cluster-forming threshold of $p < .01$ and 1,000 random permutations for forming null distributions. Clusters with test statistics exceeding the middle 95% of the empirically generated null distribution (i.e., $p < .05$, two-tailed) were considered significant.

***Cardiac Interoceptive Sensitivity, Subjective Arousal, and Anxiety***

To examine whether anxiety modulates the link between cardiac interoceptive sensitivity and subjective arousal, the magnitude of HEP effects (within a significant temporal cluster revealed by the cluster-based permutation test) was correlated with anxiety scale scores. To determine HEP effect magnitude for each participant, the average EEG signal in a significant time by channels cluster for the high arousal condition was subtracted from this average for the low arousal condition. This magnitude difference measure was correlated separately with GAD and STAI-S composite scores.

Throughout the results, we report Bayes factors characterizing evidence either in favor of or against the null hypothesis of an effect size of zero in a *t* test using the *ttestBF* package (Morey et al., 2011; Morey & Rouder, 2011; Rouder et al., 2009) in the *R* programming language (R Core Team, 2024). We use the conventional notation of $BF_{01}$ to denote evidence in favor of the null hypothesis and $BF_{10}$ to denote evidence in favor of the alternative hypothesis. We used default prior settings in all applications, which are a noninformative Jeffrey's prior placed on the variance of the normal population, and a Cauchy prior placed on the standardized effect size. We interpret Bayes factors using canonical thresholds: < 3 as anecdotal evidence, > 3 & < 10 as substantial evidence, > 10 & < 31.6 as strong evidence, > 31.6 & < 100 as very strong evidence, and > 100 as decisive evidence (Jeffreys, 1961/1998).

## Results

### Preprocessing revealed sufficient epochs and no differential impact of CFA across subjective arousal conditions

Given that the HEP is relative to the onset of a heartbeat and not to an external stimulus, enough time needs to elapse between heartbeats to cleanly measure the HEP (Petzschner et al., 2019). To prevent overlapping heartbeat events, we dropped detected R peaks that were within 700 ms of each other, resulting in an average dropout rate of 11.48% (*SD* = 14.28%) per participant. An average of 9.76 epochs per trial (*SE* = 0.20) were retained per participant, and there was substantial evidence to suggest that the number of epochs retained did not vary across subjective arousal conditions ($M_{\text{Low}} = 9.71, SE = 0.21, M_{\text{High}} = 9.82, SE = 0.20; t(50) = 1.12, p = .268, BF_{01} = 3.63$). To control for the CFA, we sought to ensure that heart rate and peak ECG amplitude did not vary across subjective arousal conditions. Comparing the average heart rate across the 10 s pre-probe period revealed substantial evidence in favor of no difference in heart rate across a median split in subjective arousal ($M_{\text{Low}} = 73.3, SE = 1.45, M_{\text{High}} = 73.6, SE = 1.45; t(50) = 1.15, p = .254, BF_{01} = 3.51$). Comparing peak ECG amplitude within the significant time window (revealed by the cluster-based permutation analysis, 328 – 364 ms post R-peak; see below) also revealed substantial evidence in favor of no difference in peak amplitude across a median split in subjective arousal ($M_{\text{Low}} = -1.91 \times 10^{-6}, SE = 6.33 \times 10^{-6}, M_{\text{High}} = -1.97 \times 10^{-6}, SE = 6.80 \times 10^{-6}; t(50) = 0.02, p = .980, BF_{01} = 6.56$). Together, these results suggest that (i) a sufficient number of epochs were being averaged per trial and (ii) the CFA was not impacting one subjective arousal condition more than the other.

**Subjective arousal is distinct from physiological arousal and other experience sampling measures**

We next examined how subjective arousal fluctuates within and between participants and how it relates to other measures collected in the experiment. Subjective arousal varied substantially between participants ($M$ = 49.1, $SD$ = 12.4), with some participants exhibiting a bimodal pattern of responding while others exhibited a unimodal, normal-shaped pattern (see *Figure 2A and Figure S1*). For some participants, subjective arousal varied widely over time, whereas others displayed more stable patterns (see *Figure* 2B).

We next examined the relationship between subjective and physiological arousal measures. Within-participant correlations between subjective arousal and heart rate ($M$ = .047, $SE$ = .025; $t(50)$ = 1.87, $p$ = .067, $BF_{01}$ = 2.0) and subjective arousal and HRV ($M$ = -.037, $SE$ = .024; $t(50)$ = 1.59, $p$ = .118, $BF_{01}$ = 2.03) exhibited anecdotal evidence of these distributions not being significantly different from zero. Within-participant correlations between subjective arousal and time-on-task ($M$ = -.012, $SE$ = .048; $t(50)$ = 0.252, $p$ = .802, $BF_{Null}$ = 6.37) exhibited substantial evidence in favor of the null hypothesis that this distribution was not different from zero (see *Figure 2C*). Interestingly, for time on task, roughly equal numbers of participants exhibited positive vs. negative correlations with subjective arousal, suggesting the mind-wandering task may have engaged arousal for some but decreased vigilance for others over time. Further, there was also substantial evidence of no difference in parieto-occipital alpha power across a median split on subjective arousal ($M_{\text{High}} = 6.15 \times 10^{-10}, SE = 1.06 \times 10^{-10}, M_{\text{Low}} = 6.54 \times 10^{-10}, SE = 1.20 \times 10^{-10}, t(50) = 1.85, p = .070, BF_{01} = 4.66$; see *Figure 2C*). Together, these results suggest our subjective arousal measure explained unique variance relative to commonly used measures of physiological arousal. However, comparing participant-level distributions of correlations to zero with one-sample *t* tests suggested the link between subjective arousal and heart rate and HRV was less conclusive ($BFs_{01} < 3$).

Having explored the relationship between subjective and physiological arousal, we next sought to more fully characterize what subjective arousal measures capture by examining its relationship with other self-reported dimensions of ongoing experience. We computed correlations between subjective arousal and all other experience-sampling items within participants and compared the resulting distributions of correlations against zero. We report the four distributions with the largest Bayes factors in favor of the alternative hypothesis (i.e., of the distributions being different from zero). There was decisive evidence that the distributions of correlations between subjective arousal and future thinking ($M = .164$, $SE = .030$, $t(50) = 5.48$, $p < .001$, $BF_{10} = 12{,}078$), deliberate thinking ($M = .174$, $SE = .038$, $t(50) = 4.47$, $p < .001$, $BF_{10} = 447$), and self-referenced thinking ($M = .146$, $SE = .034$, $t(50) = 4.22$, $p < .001$, $BF_{10} = 225$) were greater than zero. There was strong evidence that the distribution of correlations for difficulty to disengage from thoughts was greater than zero ($M = .143$, $SE = .040$, $t(50) = 3.58$, $p < .001$, $BF_{10} = 35.2$). While subjective arousal was overall related to these four dimensions of ongoing experience, the moderate strength of correlations and the standard error of correlations across participants suggests that subjective arousal carries independent variance from other dimensions of ongoing experience.

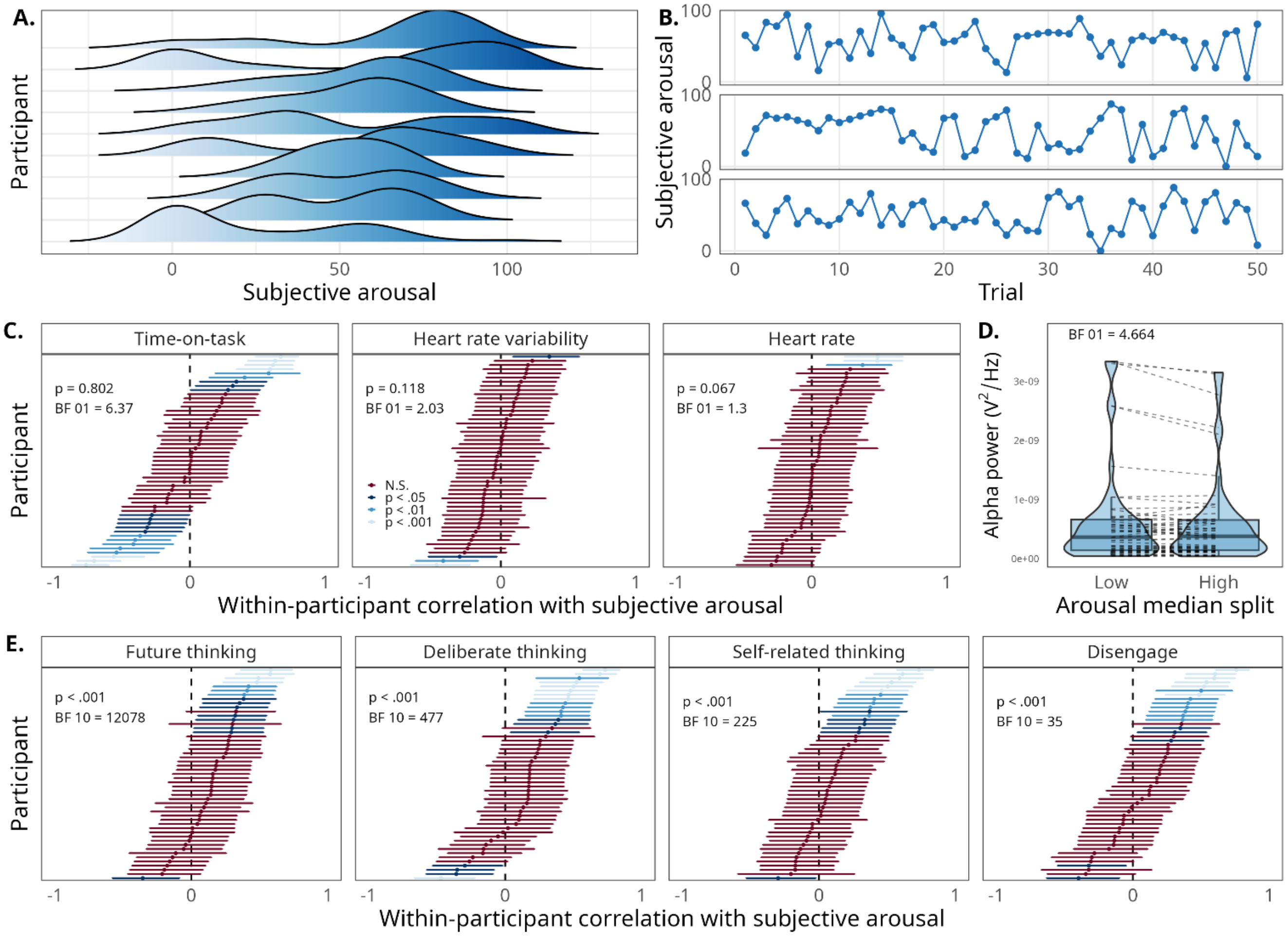

*Figure 2*. Characteristics of subjective arousal ratings and relationships with other physiological and subjective measures. (A) Gaussian kernel density estimates for subjective arousal ratings across an example subset of participants. A distribution reflects the frequency of responses on the subjective arousal scale for a particular participant across the experiment. (B) Timeseries of subjective arousal ratings across all 50 trials for an example set of 3 participants. Each point reflects a particular subjective arousal rating submitted by a participant on one trial. (C) Distributions of within-participant correlations between subjective arousal and measures of physiological arousal, including time-on-task, heart rate variability, and heart rate. Points reflect participant-level correlations; bands around points reflect 95% confidence intervals around estimated correlations. P values and Bayes factors reflect statistics from a one-sample t-test of the Fisher z-transformed distribution of participant-level correlations against a mu (i.e., null hypothesis) of zero. Color of the points and bands confers the p-value significance level (uncorrected) of the participant-specific correlation. (D) Parieto-occipital alpha power averaged across a within-participant median split in subjective arousal scores. The Bayes factor reflects evidence in favor of the null hypothesis of no difference in alpha power across subjective arousal in a paired-sample t-test. (E) Distributions of within-participant correlations between subjective arousal and the four experience sampling items that are most strongly associated with it, including future thinking, deliberate thinking, self-related thinking, and difficulty to disengage from thoughts. Plot aesthetics map to the same data aspects as in panel C.

**Lower subjective arousal is associated with greater HEP amplitude**

To assess the relationship between subjective arousal and cardiac interoceptive sensitivity, we contrasted the HEP across trials in which self-reported subjective arousal was low vs. high using a cluster-based permutation analysis. This analysis revealed one significant cluster at frontal electrodes (Fz, FC1, FC5, Fp1, F3, F7, F4, Fp2, FC2, F8) from 0.328 – 0.364 s post R peak, such that HEP amplitude was greater (i.e., more positive) on trials in which subjective arousal was rated as lower vs. higher ($p$ = .016; see *Figure 3A* and *Figure 3C*), an effect that was consistent across most participants (see *Figure 3B*). Since larger HEP amplitude is thought to reflect greater interoception (Judah et al., 2018; Pang et al., 2019; Verdonk et al., 2024), the direction of this effect implies lower subjective arousal during states of greater cardiac interoceptive sensitivity. Interestingly, the topology, time course, and direction of this effect closely replicate and extend a finding from past work, where subjective arousal was driven by an external event rather than spontaneously fluctuating at rest (Fourcade et al., 2024).

*Figure 3.* HEP effect across time, participants, and electrodes. (A) HEP grand average across the heartbeat epoch, broken down across within-participant median split in subjective arousal ratings. Timepoint zero reflects the R-peak, and colors denote the subjective arousal condition. The grey shaded region reflects the significant time window indicated by the cluster-based permutation analysis (0.328 – 0.364 s), while the colored shaded regions reflect standard errors of the mean. The EEG waveform is averaged across significant electrodes (i.e., Fz, FC1, FC5, Fp1, F3, F7, F4, Fp2, FC2, F8). (B) HEP averaged across a participant-level median split in subjective arousal collapsed across the significant time window, shown separately for each participant (dotted lines connect averages across conditions from the same participant). (C) Topographical map of *t*-values for HEP differences between high (blue) vs. low (red) levels of subjective arousal across the 0.328 – 0.364 s significant time window.

**The relationship between subjective arousal and cardiac interoceptive sensitivity depends on state anxiety**

To investigate whether increased cardiac interoceptive sensitivity during states of lower subjective arousal is linked to anxiety, we examined whether the size of the participant-level HEP effect correlated with individual difference measures in both trait (GAD) and state (STAI-S) anxiety. While there was a trending but not significant relationship between the HEP effect and GAD ($r(50) = .21$, $p = .135$), there was a significant positive correlation between the HEP effect and STAI-S ($r(29) = .4$, $p = .027$; see *Figure 4A*). In other words, the extent to which HEP amplitude was more positive for lower states of arousal was increased for those individuals who reported greater state anxiety at the outset of the experiment (*Figure 4B*).

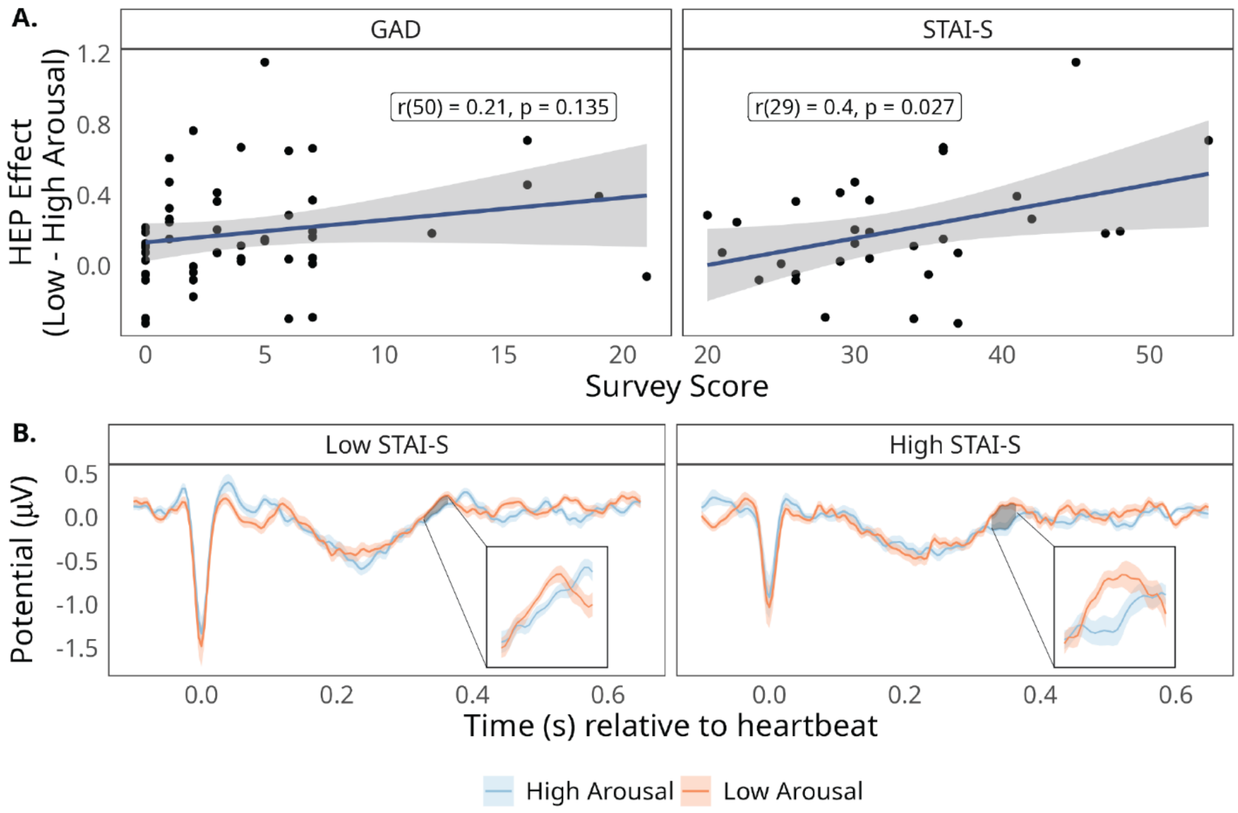

*Figure 4.* Association between HEP arousal effect and individual differences in anxiety. (A) Correlations of trait (GAD) and state (STAI-S) anxiety with individual differences in the HEP effect magnitude (higher on the y-axis reflects larger HEP amplitude in the low vs. high subjective arousal condition). (B) HEP grand average across the heartbeat epoch, broken down by within-participant median split on subjective arousal ratings and a median split on state anxiety (STAI-S) for visualization purposes only. Insets zoom in to the significant time window, indicated by the shaded grey region. Shaded colored regions reflect one standard error from the mean.

## Discussion

Here we showed that cardiac interoceptive sensitivity increases during lower spontaneous subjective arousal, with this effect amplified in individuals with high state anxiety. Subjective arousal was distinct from physiological measures like time-on-task and parieto-occipital alpha power, while its relationship with heart rate and variability was inconclusive. Subjective arousal also differed from other aspects of experience and correlated positively with future thinking, deliberate thinking, self-related thinking, and difficulty disengaging from thoughts. Cardiac interoceptive sensitivity, indexed by the HEP, showed greater amplitude just before lower subjective arousal ratings, localized at frontal electrodes (328–364 ms post-heartbeat). This effect was stronger in those with high state anxiety. Our findings offer clues into how the concept of arousal may be refined and have clinical relevance for anxiety and interoceptive dysregulation (Khalsa & Lapidus, 2016).

Arousal is widely studied but inconsistently defined (Lindquist et al., 2016; Satpute et al., 2019). Measures such as self-report, skin conductance, heart rate, respiratory frequency, and EEG alpha power often show weak or inconsistent correlations among each other (Agren, 2023; Leonidou & Panayiotou, 2022; Wenzler et al., 2017). This inconsistency has led some to conceptualize arousal as multidimensional (Reid et al., 2024), while others argue that distinct types of arousal may share underlying cortical pathways, namely the presupplementary motor areas and dorsal anterior insula (Sabat et al., 2025). These competing perspectives underscore the need to differentiate aspects of arousal. We focused on spontaneous fluctuations in self-reported subjective arousal, which showed little correlation with non-cardiac physiological measures. While subjective arousal's association with cardiac measures was examined using one-sample t tests, these did not account for within-participant uncertainty (i.e., 95% confidence intervals in Figure

2C). The fact that many confidence intervals included zero further supports the distinction between subjective arousal and cardiac physiological arousal.

Our findings lend support to the idea that arousal is a multidimensional construct with independent subcomponents (Reid et al., 2024). Although arousal has been widely studied, few investigations have examined spontaneous fluctuations in subjective arousal during wakeful rest (but see Hoemann et al., 2020). The association between subjective and physiological arousal may depend on whether attention is focused on external stimuli (Leonidou & Panayiotou, 2022), raising the possibility that self-reported arousal reflects deliberate rather than purely physiological processes. Future research should examine how spontaneous fluctuations in subjective arousal relate to attentional focus and how this interaction influences the link (or lack thereof) between subjective and physiological arousal.

Subjective arousal correlated moderately with other dimensions of ongoing experience, such as future thinking, deliberate thinking, self-related thinking, and difficulty to disengage from thoughts. Deliberate thinking, which involves effortful problem solving, has been linked to increased physiological and subjective arousal, particularly when deliberation is framed as task engagement (Kahneman, 2011; Matthews et al., 2010). Our results extend these ideas by showing a positive link between subjective arousal and deliberate thinking even when deliberate thinking is not tied to an active task. Difficulty disengaging from thoughts—often linked to repetitive negative thinking and worrying, key features of generalized anxiety disorder (Ehring & Watkins, 2008) and state anxiety (Vălenaş et al., 2017)—was also associated with subjective arousal. This finding aligns with prior research showing that self-reported trait anxiety positively correlates with physiological arousal (Hoehn-Saric & McLeod, 2000; Noteboom et al., 2001; Roos et al., 2021). Finally, subjective arousal was positively associated with both self- and future-thinking. This finding contrasts somewhat with recent work showing a negative association, at the inter-individual level, between conceptual thinking (as opposed to bodily focus) and physiological arousal (Banellis et al., 2024). Self- and future-thinking tend to co-occur and are associated with improved future mood (Ruby et al., 2013). One possibility is that subjective arousal facilitates this relationship, where engaging in self- and future-thinking enhances arousal, which in turn supports mood regulation. However, how subjective arousal and self- and future-thinking impact future mood likely depends on individual differences in anxiety. Anxiety has been associated with more vivid and detailed autobiographical future thinking (Du et al., 2022), which may amplify or distort the impact of future thinking on mood.

We investigated how interoceptive processing varies as a function of spontaneous fluctuations in subjective arousal by examining the HEP. We found that in the 328–364 ms window post-heartbeat, HEP amplitude was increased at frontal electrodes in the 10 s preceding lower subjective arousal ratings. This finding suggests that interoceptive processing is enhanced during states of lower subjective arousal. The timing and topography of this effect align with previous studies linking the HEP to arousal (Fourcade et al., 2024; Fukushima et al., 2011; Gentsch et al., 2019; Ito et al., 2019; Luft & Bhattacharya, 2015; MacKinnon et al., 2013; Marshall et al., 2017, 2018, 2020; Park et al., 2016; Schulz et al., 2013, 2015; Sel et al., 2017; Shao et al., 2011), though definitions of arousal vary widely across studies. One study particularly relevant to our work recorded electrophysiological responses while participants experienced virtual reality (VR) roller coaster rides and retrospectively rated fluctuations in subjective arousal (Fourcade et al., 2024). Their results closely parallel ours, showing increased HEP for lower subjective arousal at fronto-central electrodes within a few milliseconds of our observed time window (328–360 ms). This convergence across methodologies strengthens the link between subjective arousal and interoceptive processing indexed by the HEP. Our findings extend upon prior work that showed differences in HEP driven by external events (i.e., roller coaster videos; Fourcade et al., 2024) by revealing that such differences in HEP can also be driven by spontaneously fluctuating internal states during wakeful rest. Fourcade et al. (2024) performed source localization analyses on their EEG results to suggest that their effect, which closely parallels ours, originates from somatosensory areas. Indeed, past work using intracranial EEG has implicated a variety of brain regions in HEP activity during rest, including somatosensory cortex, insula, opercular cortex, inferior frontal gyrus, and amygdala (Babo-Rebelo, Wolpert, et al., 2016; Canales-Johnson et al., 2015; Kern et al., 2013; Park et al., 2018; Park & Blanke, 2019).

One possible reason for the increased HEP in lower subjective arousal states is that, in some contexts, reduced arousal may enhance interoception. This negative relationship between arousal and interoception can be seen in stress reduction practices, such as meditation, which both enhance interoception and reduce arousal (Hanh, 2015; Kushner & Marnocha, 2008; Nathoo, 2021; Shapiro & Carlson, 2009). In Theravada Buddhism, for example, establishing bodily calm is an explicit goal of meditation, thought to enhance sensitivity to bodily sensations and facilitate insight into impermanence (Analayo, 2018). Meditation is also empirically associated with increased interoceptive awareness, mediated through altered structural and functional neural organization, particularly in the insula (Farb et al., 2013; Hölzel et al., 2008; Luders et al., 2012). Another possibility is that higher HEP during lower subjective arousal states reflects a compensatory downregulation of interoceptive sensitivity during high-arousal states. High subjective arousal states are often subjectively experienced as anxiety (Russell, 1980, 2003), and downregulating interoceptive sensitivity may be a mechanism for regulating emotion. This possibility is supported in the present study by larger HEP effects for those higher in state anxiety, seemingly driven by reduced high-arousal HEP amplitude for high state anxiety individuals (i.e., the difference between the orange lines in *Figure 4B*). While clinical anxiety is often associated with dysregulated interoception (Khalsa & Lapidus, 2016), effects in the literature have been inconsistent (Abrams et al., 2018; Ardizzi & Ferri, 2018; Gaebler et al., 2013; Stewart et al., 2001). Our findings highlight the need to consider how momentary subjective arousal may shape the relationship between state anxiety and interoception.

This study had several limitations that can be addressed in future work. The state anxiety measure (i.e., STAI-S) was a late addition to the experiment, and as such we have data from only 30 of the 51 total participants. Our sample also contained few individuals scoring high on trait anxiety (i.e., GAD; see *Figure 4a*), which may have contributed to the absence of a correlation between HEP and trait anxiety. Future studies should replicate our findings related to state anxiety in a larger and more clinically diverse sample. A further limitation concerns the degree to which findings in our laboratory setting translate to real-world contexts. During daily life, many individuals are rarely truly at rest (i.e., devoid of prominent external stimuli or task demands). What may prove crucial in the study of mental health is understanding how spontaneously fluctuating internal states of subjective arousal interact with salient external conditions or environmental cues to influence the regulation of anxiety.

In conclusion, our findings offer advanced knowledge of the neural basis of subjective arousal and have broad implications for understanding various mental health conditions marked by emotional and interoceptive dysregulation. The interactions between HEP, subjective arousal, and anxiety that we identified suggest a potential naturally-operating mechanism in which interoception is spontaneously downregulated to facilitate emotional regulation. Further research is needed to causally test for such a mechanism, how it may be modulated, and whether evidence of this mechanism is present in other clinical populations, such as individuals with generalized anxiety disorder.

**Code Accessibility**

We will make the custom code used to analyze the electrophysiological data available upon publication.
