# Supplementary material for: Neural sensitivity to the heartbeat is modulated by fluctuations in affective arousal during spontaneous thought
Source: bioRxiv. 2025 Jul 11:2025.03.26.645574. Originally published 2025 Apr 1. Preprint. [Version 2] doi: 10.1101/2025.03.26.645574 (PMC11996350; doi:10.1101/2025.03.26.645574)
Supplement: Supplement 2 [file media-2.docx]

**Figure S2**

*Subjective Arousal Distributions by Participant*

*
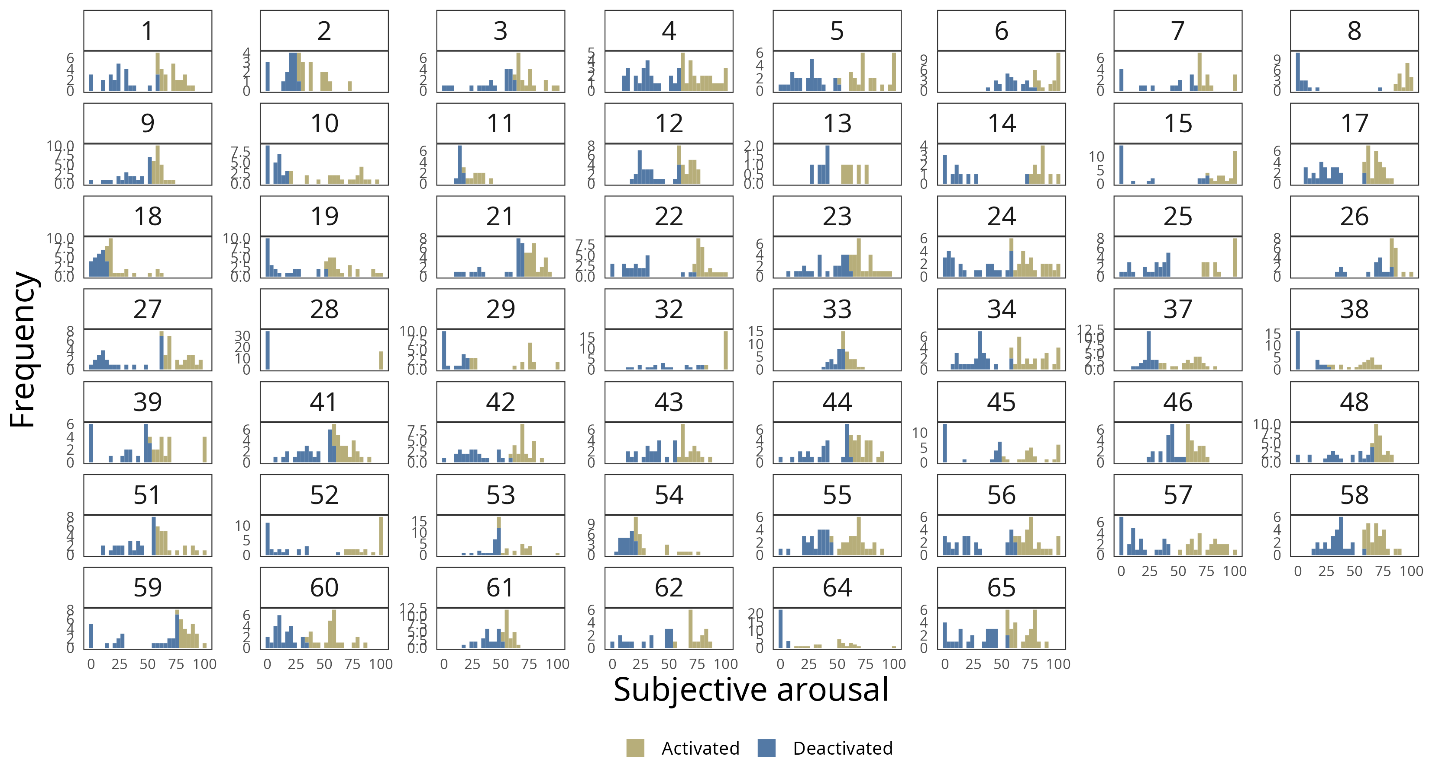
*

*Note.* Histograms reflect the observed distribution of affective arousal ratings (0 – 100) colored by a median split for each participant over the entire experiment (“Activated” maps to high affective arousal; “Deactivated” maps to low affective arousal).
