# Supplementary material for: Neural sensitivity to the heartbeat is modulated by fluctuations in affective arousal during spontaneous thought
Source: bioRxiv. 2025 Jul 11:2025.03.26.645574. Originally published 2025 Apr 1. Preprint. [Version 2] doi: 10.1101/2025.03.26.645574 (PMC11996350; doi:10.1101/2025.03.26.645574)
Supplement: Supplement 3 [file media-3.docx]

**Figure S3**

*Results of surrogate cluster control test*

*
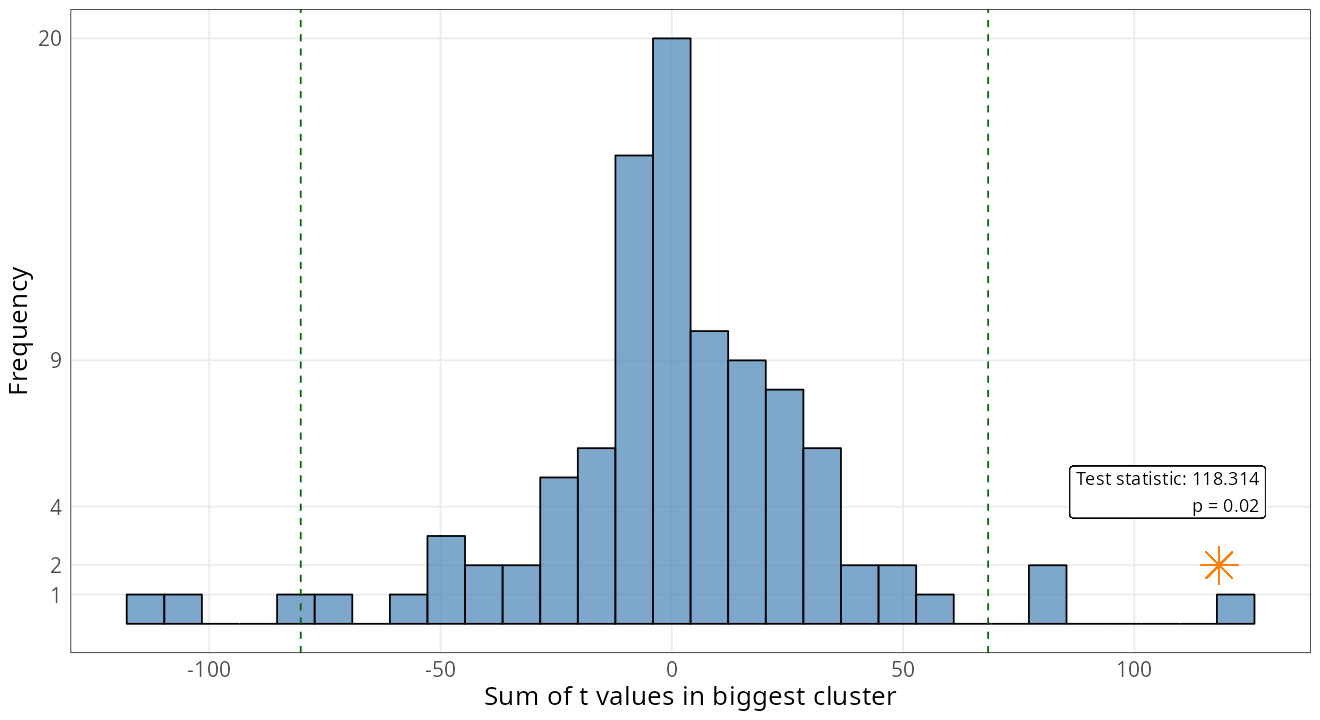
*

*Note.* The summed *t* value derived from summarizing epochs time-locked to heartbeats (118.314) was significantly greater (*p* = .02) than a null distribution of summed *t* values obtained from summarizing epochs that were randomly shuffled in time (i.e., decoupled from heartbeats).
